# Modeling the Clockwork of Bone: A Narrative Review of Experimental Approaches to Circadian Rhythm in Bone Metabolism

**DOI:** 10.3390/ijms27125167

**Published:** 2026-06-07

**Authors:** Xiang Gao, Xinyuan Cai, Andreas K. Nussler

**Affiliations:** Siegfried Weller Research Institute, BG Unfallklinik Tuebingen, Department of Trauma and Reconstructive Surgery, University of Tuebingen, Schnarrenbergstr. 95, D-72076 Tuebingen, Germany; xianggao23@gmail.com (X.G.); xinyuancai97@gmail.com (X.C.)

**Keywords:** circadian rhythm, bone remodeling, in vivo model, ex vivo model, in vitro model, osteoblasts, osteoclasts

## Abstract

Circadian rhythms are fundamental regulators of skeletal homeostasis, coordinating osteoblast and osteoclast activity through tightly controlled temporal programs. Disruption of these rhythms, whether through environmental misalignment or genetic perturbation of core clock components, alters bone formation, enhances resorption, and contributes to skeletal fragility. This review synthesizes current knowledge on circadian regulation of bone biology across in vivo, ex vivo, and in vitro model systems, highlighting how each platform reveals distinct aspects of rhythmic gene expression, cellular function, and tissue-level remodeling. We critically evaluate the strengths and limitations of these models, outline key controversies such as the interpretation of global clock-gene knockouts, and discuss the emerging relevance of human-derived systems including iPSC-based models, organoids, and microphysiological “bone-on-chip” platforms. Integrative approaches that combine multiple model systems provide the most reliable framework for understanding circadian control of bone and for identifying targets for chronotherapeutic intervention. Advancing human-relevant models and refining temporal experimental design will be essential for translating circadian biology into clinical strategies for metabolic bone diseases.

## 1. Introduction

Circadian rhythms are fundamental regulators of physiological homeostasis, coordinating metabolic, endocrine, immune, and behavioral processes across multiple organ systems [1,2,3,4,5,6,7]. Recent evidence has demonstrated that these rhythms also play a direct and mechanistically relevant role in skeletal biology, influencing the temporal coordination of osteoblast, osteoclast, and osteocyte activity [8,9,10,11,12]. Despite growing interest in this field, the mechanistic pathways linking circadian oscillations to bone remodeling remain only partially defined, and existing studies often differ in experimental design, model selection, and temporal resolution. Disruptions of circadian homeostasis arising from shift work, jet lag, aging, or genetic alterations have been associated with impaired bone mineral density, delayed fracture healing, and increased skeletal fragility [10,11,12,13,14]. At the molecular level, core clock genes such as Bmal1, Clock, Per1/2, and Cry1/2 exhibit rhythmic expression in bone tissue and regulate key markers of bone formation and resorption, including RANKL, OPG, CTX, PINP, ALP, and TRAP [15,16]. Misalignment of these pathways may alter the temporal coupling of bone formation and resorption, thereby contributing to metabolic bone disorders. To provide a more integrated and mechanistically informative perspective, this review critically evaluates the experimental approaches used to investigate circadian regulation in bone metabolism, including in vivo, ex vivo, and in vitro systems. We highlight their respective strengths, limitations, and translational relevance, and we synthesize key findings that illuminate how circadian rhythms shape skeletal homeostasis.

In response to recent advances in the field, we further incorporate emerging human-relevant platforms, 3D co-culture systems, and microphysiological models, and we discuss unresolved controversies, methodological challenges, and opportunities for chronotherapeutic applications.

## 2. Search Strategy

A comprehensive literature search was conducted using the PubMed database to identify studies investigating the interplay between circadian rhythms and bone biology.

To enhance methodological transparency and reproducibility, we refined the search workflow and explicitly defined the review as a narrative review with systematic elements.

The search covered the period from July 2005 to July 2025 and used the keywords “circadian rhythm”, “bone”, and “model”. All article types, including original research and reviews, were considered. The initial search yielded 165 records.

The search covered the period from July 2005 to July 2025 and used the keywords “circadian rhythm”, “bone”, and “model”. All article types, including original research and reviews, were considered. The initial search yielded 165 records. Inclusion criteria were: (1) explicit investigation of circadian regulation in bone or bone-related cells; (2) use of an identifiable experimental model (in vivo, ex vivo, or in vitro); and (3) availability of full text in English. Exclusion criteria included (1) studies addressing only circadian biology without skeletal relevance; (2) studies on bone biology without circadian endpoints; and (3) non-English publications.

**Figure 1:**
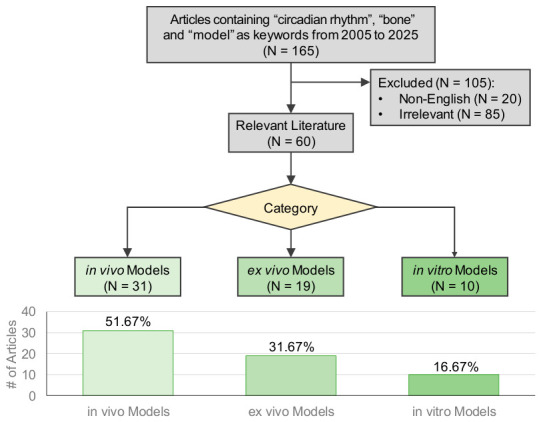
This PRISMA-style diagram summarizes the structured search workflow used to identify studies investigating circadian regulation in bone biology. The initial PubMed search (July 2005–July 2025) using the keywords “circadian rhythm”, “bone”, and “model” yielded 165 records. After removal of duplicates and application of predefined inclusion criteria (studies addressing circadian rhythm, bone biology, and an identifiable experimental model), 60 articles were retained for qualitative synthesis. Exclusion criteria included non-English publications, studies lacking full text, and studies addressing only one or two of the three core topics. The final dataset comprised 31 in vivo studies (51.67%), 19 ex vivo studies (31.67%), and 10 in vitro studies (16.67%). This workflow reflects the narrative-review design with systematic elements and provides transparency regarding study identification, screening, and eligibility assessment.

After screening, 60 articles met the criteria, comprising 48 original research papers and 12 reviews. Duplicate entries were removed. A PRISMA-style flow diagram has been added to illustrate the search workflow and study selection process (Figure 1).

## 3. Model Systems

### 3.1. In Vivo Model Systems

In vivo models are indispensable for exploring the interplay between circadian rhythms and bone metabolism. By preserving the physiological context of a living organism, these models enable the study of bone remodeling processes under the influence of systemic factors such as hormonal signaling, immune responses, neural regulation, and mechanical loading. Rodents, particularly mice, are commonly employed in this field due to their genetic tractability and physiological similarities to the human circadian system.

Murine in vivo systems can be broadly categorized into (1) environmental disruption models and (2) genetic manipulation models, each providing distinct insights into circadian regulation of skeletal physiology.

Environmental disruption models frequently employ jet-lag–like protocols, such as shifting the light–dark cycle by eight hours every three days [17] or exposing animals to weekly alternating bright/dim cycles [18,19]. While continuous inversion of the bright–dim cycle primarily exacerbates inflammation-associated bone degradation [19], repeated weekly shifts induce pronounced trabecular and cortical bone loss, highlighting the sensitivity of skeletal tissue to chronic circadian misalignment [18,19]. Importantly, gestational exposure to circadian disruption has been shown to impair bone development in offspring, suggesting transgenerational effects of rhythm perturbation [17]. Similar findings have been reported in periodontal disease models, where circadian disruption exacerbates alveolar bone loss and alters macrophage-mediated inflammatory responses [20,21,22,23].

To counter these effects, therapeutic interventions have been explored, notably melatonin, a hormone closely linked to circadian regulation. In experimental models, melatonin has been reported to restore rhythmic stability and attenuate bone degeneration, suggesting potential utility as an adjunctive strategy for conditions such as osteoporosis—for review see [24,25]. However, the magnitude and timing of melatonin’s skeletal effects remain variable across studies, underscoring the need for standardized temporal dosing paradigms.

Genetic knockout models provide deeper insight into the molecular basis of skeletal health. For example, removal of BMAL1, a core clock gene, has been shown to interfere with osteoblast maturation [26], resulting in reduced bone formation and an osteoporosis-like phenotype—for review see [27]. However, global Bmal1 knockouts also exhibit premature aging, sarcopenia, altered feeding behavior, and hormonal dysregulation, complicating the interpretation of bone-specific phenotypes. To address this, conditional knockouts (e.g., Bmal1fl/fl; Ocn-Cre or Bmal1fl/fl; LysM-Cre) have been developed to isolate cell-type-specific circadian effects on osteoblasts and osteoclasts.

In addition to mouse models, researchers have employed other species to investigate circadian effects on bone metabolism, including laying hens. Dietary phosphate feeding regimens showed circadian effects on eggshell deposition and thus eggshell quality [28]. Simultaneously, medullary bone samples collected showed inverse regulation of bone metabolism (eggshell strengthening led to bone weakening), as detected by osteoblast and osteoclast function [28]. These findings illustrate how circadian-driven mineral mobilization can differentially affect skeletal compartments.

A major strength of in vivo systems is their capacity to preserve endogenous circadian synchronization across multiple tissues. This feature enables the study of complex intercellular and inter-organ interactions and facilitates modeling of bone-related pathologies such as osteoporosis, diabetes, and periodontitis [6,21,23,25,29,30]. Advanced genetic tools, including transgenic reporter lines (e.g., Per1: Luc mice), further enhance the precision of these investigations by enabling real-time monitoring of clock gene activity in bone cells [31,32].

**Figure 2:**
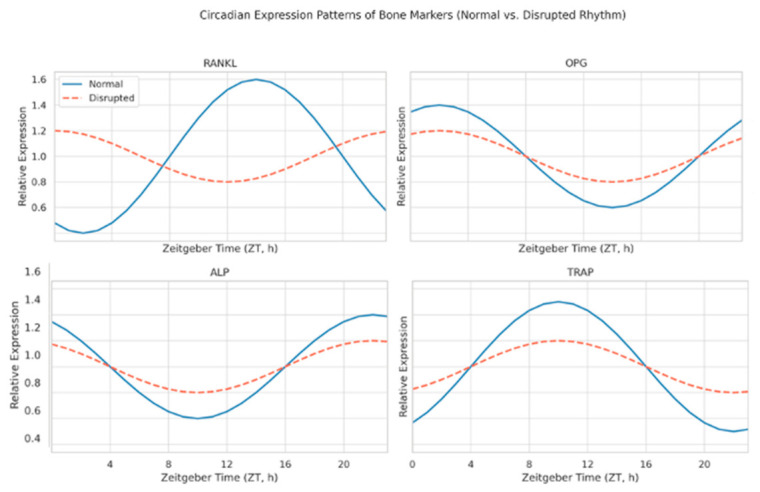
Conceptual summary of circadian expression patterns of bone markers. The figure depicts the 24 h Zeitgeber Time (ZT) rhythmicity of major bone-remodeling markers during physiological (blue) and disrupted (red, dashed) circadian conditions. The oscillatory patterns are adapted from the findings of Dovio et al. [35] and Diemar et al. [16]. Under normal circadian alignment, RANKL, OPG, ALP, and TRAP display coordinated, high-amplitude rhythms that reflect tightly regulated bone turnover. Circadian disruption leads to attenuated oscillatory amplitude, altered phase timing, and loss of rhythmic coherence, indicating compromised temporal control of bone formation and resorption. The schematic diagram was visualized using ChatGPT (5.0). Xiang Gao (2025).

Rhythmic bone cell function is typically assessed by measuring serum biomarkers of bone formation and resorption, as well as regulatory markers [16,33,34] (Figure 2). Bone formation markers: PINP, PICP, osteocalcin (OCN), bone-specific ALP, Bone resorption markers: CTX, NTX, CAII, TRAP, cathepsin K, Bone metabolism regulatory markers: RANKL, OPG, fibroblast growth factor 23, leptin.

**Figure 3:**
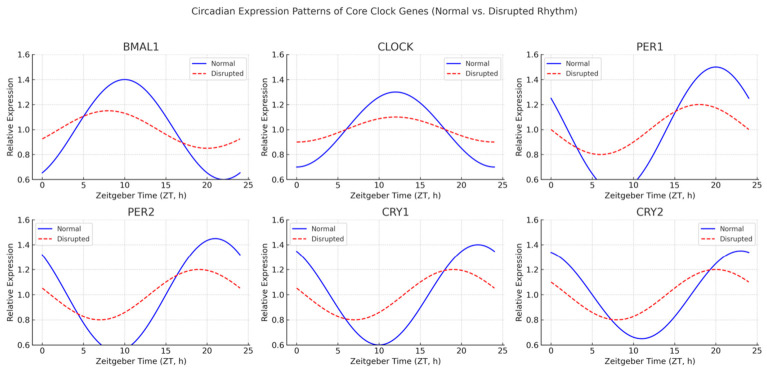
Conceptual summary of circadian expression patterns of core clock genes. The figure depicts the 24 h ZT oscillatory profiles of the core clock genes *Bmal1*, *Clock*, *Per1*, *Per2*, *Cry1*, and *Cry2* in mice during normal (blue) and disrupted (red, dashed) circadian conditions. The expression patterns are adapted from Schilperoort et al. [18] and Zvonic et al. [15]. Under physiological entrainment, these genes exhibit coordinated, high-amplitude rhythms that maintain temporal order within peripheral tissues. Circadian misalignment results in attenuated oscillations, altered phase timing, and reduced rhythmic coherence, which may impair downstream pathways involved in bone remodeling. The schematic diagram was visualized using ChatGPT (5.0). Xiang Gao (2025).

Additionally, expression levels of core circadian genes (Bmal1, Clock, Per1/2, Cry1/2) can be quantified in bone tissue using qRT-PCR, in situ hybridization, or bioluminescence imaging. Hormones with circadian variation, such as melatonin, are also monitored through timed blood sampling to evaluate endocrine influences on bone remodeling—for review see [24,25,36]. The oscillatory behavior of core clock genes in bone tissue is illustrated in Figure 3.

Despite their advantages, in vivo models face several challenges. Their systemic complexity introduces confounding factors (e.g., altered locomotor activity, feeding behavior, hormonal rhythms), making it difficult to attribute skeletal changes solely to circadian mechanisms. Moreover, these studies are resource-intensive and must comply with rigorous ethical standards. Collecting samples at multiple time-points, which is essential for circadian analysis, is technically demanding and may itself disrupt physiological rhythms. Furthermore, anesthesia, euthanasia timing (ZT vs. CT), and stress responses can significantly bias gene expression and biomarker measurements, a methodological issue often underreported in the literature.

**Figure 4:**
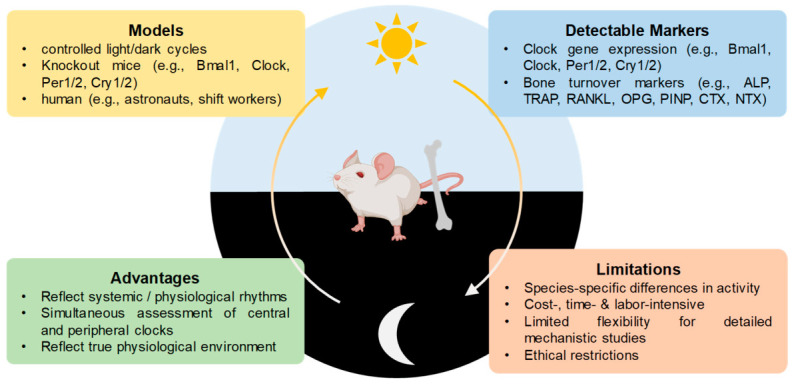
Visualization of in vivo models. In vivo models are instrumental in elucidating the dynamic interplay between circadian rhythms and bone remodeling processes. These models typically involve rodents, especially mice, maintained under controlled light–dark cycles or genetically engineered to lack key circadian genes such as *Bmal1*, *Clock*, *Per1/2*, *and Cry1/2*. Such setups allow researchers to investigate the systemic and tissue-specific effects of circadian disruption on skeletal physiology. However, animal models also have their limitations, such as high costs and ethical concerns. Image was created in BioRender. Xiang Gao (2025) (https://app.biorender.com/illustrations/69ca989269f3c9dfd83ad093, accessed on 1 June 2026).

In summary, in vivo models remain the cornerstone of circadian bone research due to their physiological relevance, but careful experimental design and temporal control are essential to avoid misinterpretation of systemic versus bone-intrinsic circadian effects (Figure 4).

### 3.2. Ex Vivo Model Systems

Ex vivo models represent an intermediate platform between in vivo and in vitro systems for investigating circadian regulation in bone biology. These models typically involve the extraction of intact tissues such as bone slices, periodontal segments, or intervertebral discs from animal or human sources at defined circadian time points, followed by short-term culture under controlled environmental conditions [37]. This strategy allows the investigation of intrinsic rhythmic activity in bone cells outside the organism, while maintaining native tissue architecture and intercellular interactions.

Although recent literature reports on circadian studies using transgenic *Per1: Luc* mice are limited, this model remains one of the most widely adopted ex vivo systems for circadian research. In *Per1: Luc* and *Per2: Luc* mice, the real-time expression of the core clock genes *Per1* and *Per2* can be visualized through bioluminescence imaging in bone explants [31,38,39]. These studies have demonstrated that peripheral tissues, including bone, retain autonomous circadian oscillations ex vivo for several days, allowing time-resolved analysis of clock gene expression and bone cell function. However, oscillatory amplitude typically dampens after 48–72 h, reflecting the absence of systemic synchronizing cues.

Beyond luminescent reporters, ex vivo systems allow the quantification of various circadian genes and bone-related markers. Clock genes such as *Bmal1*, *Clock*, *Per1/2*, *NR1D1*, and *Cry1/2*, as well as osteoblast and osteoclast markers, are commonly assessed using qRT-PCR, ELISA, Western blot, or dot blot techniques [40,41]. Specific bone markers secreted into the culture supernatant may also be detected by ELISA or dot blot [41]. Extracellular calcium matrix and bone tissue morphology are routinely assessed by (immuno-histological staining, scanning electron microscopy, or Raman spectroscopy [38,40,42,43]. These multimodal readouts enable the simultaneous evaluation of circadian gene oscillations and functional bone remodeling endpoints. Key circadian readouts commonly used in bone research are summarized in Table 1.

In addition to classical ex vivo methodologies, ex vivo models frequently incorporate transgenic mice or rats as cell or tissue sources, offering valuable translational insights into the potential clinical consequences of circadian misalignment on bone physiology. One study showed that surgical procedures were performed on the hind limbs of transgenic mice to induce femoral fractures, followed by external fixation of the fracture site. After a defined period of stabilization, the fractured femoral tissue was excised and subsequently cultured in vitro. The expression of circadian rhythm-related genes in the bone tissue was first assessed under baseline culture conditions. Thereafter, stimulating factors such as parathyroid hormone (PTH) were introduced, and gene expression was reevaluated to determine the regulatory effects of these stimuli on circadian gene expression in bone. The results showed that PTH may have a potential role in promoting fracture healing [38]. This approach illustrates how ex vivo systems can be used to dissect time-dependent responses to therapeutic interventions.

In another ex vivo experiment, the role of Rev-erbα in growth plate cartilage was investigated. Metatarsal tissue was isolated from mice and cultured under controlled conditions, after which a Rev-erbα antagonist was introduced into the culture medium. Subsequent analyses assessed bone tissue proliferation, differentiation, and mineralization. The findings demonstrated that inhibition of Rev-erbα suppressed growth plate development and longitudinal elongation of metatarsals, primarily through upregulation of the MAPK–ERK1/2 signaling pathway [40]. These results highlight the potential of ex vivo systems to evaluate pharmacological modulation of circadian regulators in bone.

Ex vivo systems offer distinct advantages for circadian research in bone: they reduce the systemic variability inherent in whole-animal models, permit high-resolution temporal sampling, and preserve native tissue architecture and cell–cell interactions that are absent in traditional monolayer cultures. Nonetheless, these models are constrained by the lack of systemic regulatory inputs, such as hormonal and neural signals, and by the limited viability of tissue outside the organism, which restricts long-term rhythmic analysis [44]. Moreover, circadian oscillations in ex vivo bone tissue typically dampen rapidly, and synchronization protocols (e.g., serum shock, temperature cycles, or pharmacological cues) may be required to maintain rhythmicity. These methodological limitations must be carefully considered when interpreting ex vivo circadian data.

**Figure 5:**
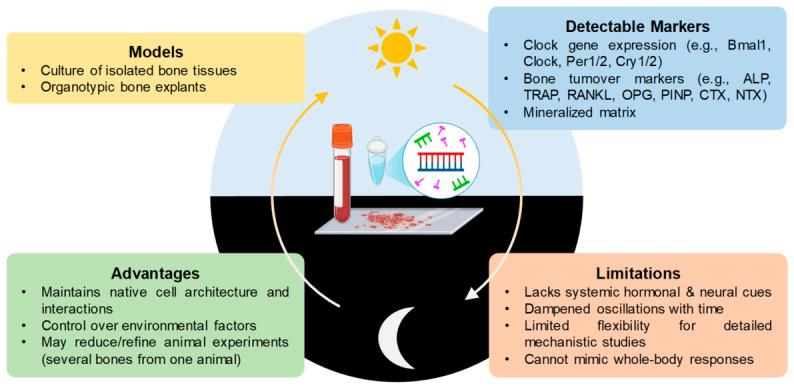
Visualization of ex vivo models. Ex vivo models are useful for studying circadian regulation in bone tissues while preserving some native structure and cell–cell interactions. These models typically involve culturing bone or bone marrow-derived samples to monitor time-dependent changes in clock genes (e.g., *Bmal1*, *Clock*, *Per1/2*, *Cry1/2*) and bone markers like PICP, PINP, ALP, CTX, TRAP, OPG, RANKL, OCN, and NTX. Common methods include qRT-PCR, ELISA, Western blot or dot blot, luciferase assays, and immunostaining. While these systems offer precise control over environmental factors, the absence of systemic cues may limit sustained circadian rhythms compared to in vivo models. Image was created in BioRender. Xiang Gao (2025) (https://app.biorender.com/illustrations/69ca989269f3c9dfd83ad093, accessed on 1 June 2026).

Taken together, ex vivo models provide a powerful yet temporally constrained platform for studying intrinsic circadian properties of bone tissue. When combined with genetic manipulation, pharmacological perturbation, and advanced imaging techniques, they offer valuable mechanistic insights that complement both in vivo and in vitro approaches (Figure 5).

### 3.3. In Vitro Model Systems

In vitro systems provide a flexible and controlled framework for exploring the molecular and cellular dynamics of circadian rhythms in bone biology. These models typically involve culturing isolated bone cell types such as osteoblasts, osteoclasts, or mesenchymal stromal cells (MSCs) under well-defined laboratory conditions. This setup enables researchers to examine intrinsic circadian oscillations and functional responses without interference from systemic physiological factors. In vitro approaches can be broadly divided into mono-culture, co-culture, and advanced 3D or microphysiological systems, each offering distinct advantages for dissecting cell-intrinsic circadian mechanisms.

#### 3.3.1. Simulating Circadian Rhythms In Vitro

To mimic circadian fluctuations in vitro, cells must be synchronized using external cues known as zeitgebers. Commonly employed zeitgebers include serum starvation followed by serum shock (e.g., 50% fetal bovine serum for 2 h) [45] or PTH stimulation [38]. Once synchronized, the oscillatory expression of core clock genes such as *Bmal1*, *Clock*, *Per1/2*, and *Cry1/2* can be tracked over 24 to 72 h using qRT-PCR, ELISA, Western blot, dot blotting, or bioluminescence-based real-time reporting in luciferase-tagged cell lines or primary cultures [46]. However, circadian oscillations in vitro typically dampen rapidly, often within 2–3 cycles, due to the absence of systemic entrainment cues. This limitation necessitates repeated synchronization or the use of perfusion-based culture systems to maintain rhythmicity.

#### 3.3.2. Functional Insights into Circadian Gene Regulation

In vitro platforms are instrumental in uncovering how circadian genes influence bone cell behavior. For example, knockdown or gene editing of Bmal1 or Clock in osteoblasts has been shown to influence apoptosis, proliferation, differentiation, and matrix mineralization. These effects are often mediated through pathways such as *Wnt*, *Sirt1*, *MAPK*, and *ERK1/2* [47,48,49,50]. In osteoclast precursors derived from Bmal1-deficient mice, studies have reported an increase in bone-loss phenotype [51], partly due to altered expression of key osteoclast markers such as TRAP and carbonic anhydrase II (CAII) [31]. Recent CRISPR-based screens have further identified additional circadian regulators in osteoblasts and osteoclasts, highlighting the complexity of clock-controlled transcriptional networks in bone.

One noteworthy investigation employed human periodontal ligament fibroblast (PDLF)-like cells cultured under mechanical stress, supplemented with 10% fetal calf serum and varying concentrations of melatonin, a hormone known to modulate circadian rhythms. The study revealed that PDLFs differentiated into osteoclast-like cells, which was suggested to be mediated by melatonin-induced activation of the core clock gene Bmal1 [52]. Such findings underscore the potential of in vitro systems to model both mechanotransduction and hormonal entrainment of circadian pathways in bone cells.

#### 3.3.3. Advanced Co-Culture and 3D Systems

**Figure 6:**
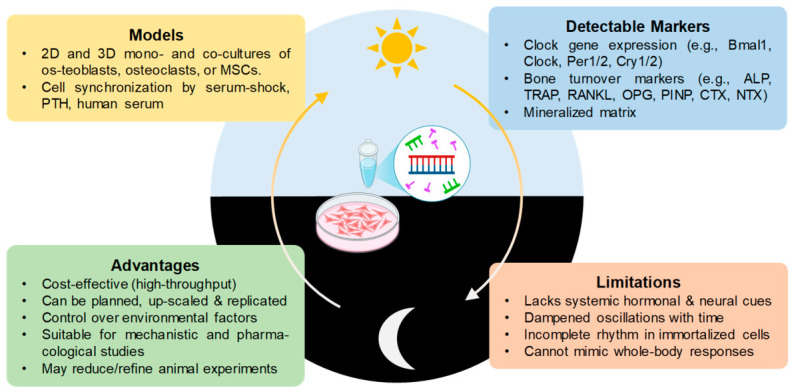
Visualization of in vitro models. In vitro models rely on cultured osteoblasts, osteoclasts, or their co-cultures to investigate circadian regulation at the cellular level. Serum shock is commonly used to synchronize rhythms, allowing analysis of clock genes (*Bmal1*, *Clock*, *Per1/2*, *Cry1/2*) and functional markers such as mitochondrial activity, ALP, TRAP, CAII, and Alizarin Red. These systems are low-cost, scalable, and highly controllable, ideal for mechanistic and drug screening studies. However, they lack systemic inputs, and circadian rhythms may weaken over time, especially in immortalized cell lines. Image was created in BioRender. Xiang Gao (2025) (https://app.biorender.com/illustrations/69ca989269f3c9dfd83ad093, accessed on 1 June 2026).

Advanced co-culture systems, including osteoblast–osteoclast co-cultures, MSC-derived 3D constructs, and microphysiological “bone-on-chip” platforms, provide more physiologically relevant environments for studying circadian regulation. These systems allow researchers to observe temporal gene expression and dynamic interactions under controlled conditions, providing a more integrated perspective on circadian regulation in bone. 3D scaffolds and perfused microfluidic systems can sustain circadian oscillations longer than traditional monolayers, reduce damping, and enable high-resolution temporal sampling of both gene expression and functional remodeling markers (Figure 6).

#### 3.3.4. Limitations of In Vitro Systems

Despite their advantages, in vitro models face several limitations. Traditional 2D cultures lack the mechanical, biochemical, and spatial cues required for physiological circadian entrainment. Moreover, immortalized cell lines often exhibit weaker or unstable circadian rhythms compared to primary human cells, raising concerns about model validity. Additionally, the absence of systemic hormonal rhythms (e.g., melatonin, cortisol) limits the translational relevance of in vitro findings unless supplemented experimentally.

Nevertheless, when combined with genetic manipulation, pharmacological perturbation, and advanced culture technologies, in vitro systems remain indispensable for mechanistic circadian research in bone biology. A comparative summary of the strengths, limitations, and typical readouts of each model system is provided in Table 2.

## 4. Discussion

The present review provides a comprehensive synthesis of experimental approaches used to investigate circadian regulation in bone biology. By evaluating in vivo, ex vivo, and in vitro systems, we highlight how each model contributes unique but complementary perspectives on the temporal regulation of skeletal homeostasis.

Across model systems, a consistent theme emerges, such as circadian rhythms exerting a profound influence on bone remodeling, affecting osteoblast differentiation, osteoclast activity, and the balance between bone formation and resorption [10,11,12,15,16]. Environmental disruption models demonstrate that misalignment of light-dark cycles leads to measurable bone loss [17,18,19], while genetic models reveal that core clock genes such as Bmal1, Clock, Per, and Cry regulate key pathways involved in osteogenesis and osteoclastogenesis [18,26,27,31,53]. However, the magnitude and direction of these effects vary across studies, reflecting differences in model design, sampling time, and species-specific physiology.

Ex vivo systems confirm that bone tissue retains intrinsic circadian oscillations [31,38,39], yet rapid damping of rhythms and the absence of systemic entrainment cues limit long-term analysis [44]. In vitro models provide mechanistic resolution, enabling targeted manipulation of clock genes and signaling pathways [47,48,49,50]. Nevertheless, the circadian robustness of immortalized cell lines remains questionable, and primary human cells exhibit donor-dependent variability that complicates interpretation [54].

A key insight from this review is that no single model can fully capture the complexity of circadian regulation in bone. In vivo models provide physiological relevance but are confounded by systemic factors such as hormonal rhythms, feeding behavior, and locomotor activity [6,21,23,25,29,30]. Ex vivo models preserve tissue architecture but lack endocrine and neural inputs [37,40,41,42,43]. In vitro models offer mechanistic precision but oversimplify the biological environment [45,46]. Integrative approaches that combine these systems are therefore essential for generating reliable and translationally meaningful conclusions.

Finally, the translational implications of circadian bone biology remain underexplored. While clinical studies suggest associations between circadian disruption and skeletal fragility [14,16,33,55,56], the potential for chronotherapeutic interventions such as time-optimized dosing of osteoporosis medications or melatonin supplementation requires further investigation [24,25,29].

## 5. Limitation

This review has several limitations that should be acknowledged. First, although we aimed to provide a comprehensive overview, the rapidly expanding nature of circadian research means that newly published studies may not be captured. Second, the heterogeneity of experimental designs across studies, including differences in sampling intervals, zeitgeber conditions, genetic backgrounds, and analytical methods, limits direct comparability [17,18,19,31,38,40]. Standardized protocols for circadian bone research are lacking, making it difficult to draw definitive conclusions across model systems.

Third, many studies rely on rodent models, which exhibit nocturnal behavior and species-specific differences in bone turnover rhythms [18,19,28]. This raises concerns about the translatability of preclinical findings to humans, particularly in the context of clinical chronotherapy. Fourth, ex vivo and in vitro systems are constrained by rapid damping of circadian oscillations, limited tissue viability, and the absence of systemic hormonal cues [38,39,40,44,45]. These limitations may lead to underestimation of circadian effects or misinterpretation of rhythmic patterns.

Finally, the field lacks consensus on appropriate statistical methods for analyzing circadian data. While tools such as cosinor analysis, JTK_CYCLE, and CircaCompare are increasingly used, differences in analytical pipelines can yield divergent interpretations of rhythmicity [57].

## 6. Controversies, Human-Relevant Models, and Integrative Approaches

### 6.1. Controversies and Unresolved Questions

Despite substantial progress, several controversies remain unresolved. In reality, skeletal circadian phenotypes vary widely depending on species, genetic background, environmental conditions, and methodological choices.

A major point of debate concerns the interpretation of global clock-gene knockout models. These models exhibit systemic alterations, including metabolic dysregulation, hormonal imbalance, reduced locomotor activity, and premature aging that complicate th attribution of skeletal phenotypes to bone-intrinsic circadian mechanisms [26,27,51]. Whether bone phenotypes arise from intrinsic clock disruption or secondary systemic effects remains an open question.

Another unresolved issue is the stability of circadian rhythms in primary human bone cells. Emerging evidence suggests that human osteoblasts and osteoclasts may exhibit weaker or more variable oscillations than immortalized cell lines [16,58]. Donor-specific factors such as age, sex, metabolic status, and medication history may further influence rhythmicity.

### 6.2. Human-Relevant Models

Human-relevant models are essential for bridging the translational gap. Bone biopsies and surgical waste samples provide direct access to human skeletal tissue [16,33,55,56], but sampling time is difficult to control, and donor variability introduces significant noise. iPSC-derived osteoblasts and osteoclasts offer standardized differentiation and patient-specific modeling, yet their circadian robustness and maturation state require further validation [58].

Emerging platforms such as bone organoids and microphysiological ‘bone-on-chip’ systems enable multi-cellular interactions, mechanical stimulation, and controlled entrainment. These systems hold promise for modeling human-specific circadian dynamics but remain technically demanding and not yet widely adopted.

### 6.3. Integrative Approaches Across Model Systems

Circadian regulation of bone is a multi-scale process involving molecular, cellular, tissue-level, and systemic interactions. Integrative workflows that combine in vivo, ex vivo, and in vitro models can overcome the limitations of individual systems.

For example:

- In vivo models identify physiologically relevant rhythms and systemic influences [17,18,19,29].
- Ex vivo systems test whether these rhythms persist in isolated tissue [31,38,39,40].
- In vitro models dissect molecular pathways and enable targeted manipulation [45,46,47,48,50,51].

Such multi-tiered strategies reduce model-specific bias, enhance reproducibility, and support the development of translationally relevant chronotherapeutic interventions.

## 7. Conclusions

Circadian rhythms play a fundamental role in coordinating bone remodeling, influencing osteoblast and osteoclast activity, and shaping skeletal homeostasis across molecular, cellular, and systemic levels. Evidence from in vivo, ex vivo, and in vitro studies demonstrates that both environmental circadian disruption and genetic perturbation of core clock components can impair bone formation, enhance resorption, and alter tissue-level remodeling dynamics. Although each model system provides unique insights, their inherent limitations underscore the need for integrative, multi-scale approaches to fully understand circadian control of bone biology. Advancing human-relevant platforms and refining temporal experimental design will be essential for translating these findings into clinical strategies, including the development of chronotherapeutic interventions for metabolic bone diseases.

## Figures and Tables

**Table 1:** Circadian Readouts Commonly Used in Bone Research.

Marker/Gene	Cell Type/Process	Interpretation
PINP, PICP	Osteoblast activity	Bone formation
CTX, NTX	Osteoclast activity	Bone resorption
RANKL/OPG	Osteoblast–osteoclast coupling	Resorption/formation balance
*Bmal1*, *Clock*, *Per1/2*, *Cry1/2*	Core clock genes	Circadian rhythmicity

**Table 2:** Comparative Overview of Model Systems.

Model System	Strengths	Limitations	Typical Readouts
In vivo	Physiological relevance; systemic entrainment; hormonal and neural inputs	Confounding systemic factors; species differences; time-point sampling difficult	Serum markers (PINP, CTX), bone histomorphometry, gene expression
Ex vivo	Preserves tissue architecture; high temporal resolution	Rapid damping; no systemic cues; limited viability	*Per1/Per2* bioluminescence, qPCR, histology
In vitro	Mechanistic precision; genetic manipulation; controlled entrainment	Oversimplified environment; weak rhythms in some cell lines	qPCR, Western blot, luciferase reporters

## Data Availability

No new data were created or analyzed in this study. Data sharing is not applicable to this article.

## Abbreviations

ALP	alkaline phosphatase
CAII	carbonic anhydrase II
CTX	collagen type I C-telopeptide
ERK	extracellular signal-regulated protein kinases
MSC	mesenchymal stromal cells
NTX	n-terminal cross-linked telopeptide of type I collagen
MAPK	mitogen-activated protein kinase
OPG	osteoprotegerin
OCN	osteocalcin
PICP	carboxy-terminal propeptide of type I procollagen
PINP	procollagen type I N-terminal propeptide
PTH	parathyroid hormone
PDLF	periodontal ligament fibroblast
qRT-PCR	quantitative reverse-transcription polymerase-chain reaction
RANKL	receptor activator of nuclear factor kappa-Β ligand
TRAP	tartrate-resistant acidic phosphatase
ZT	zeitgeber time
